# Patients with chronic migraine without history of medication overuse are characterized by a peculiar white matter fiber bundle profile

**DOI:** 10.1186/s10194-020-01159-6

**Published:** 2020-07-18

**Authors:** Gianluca Coppola, Antonio Di Renzo, Emanuele Tinelli, Barbara Petolicchio, Cherubino Di Lorenzo, Vincenzo Parisi, Mariano Serrao, Valentina Calistri, Stefano Tardioli, Gaia Cartocci, Francesca Caramia, Vittorio Di Piero, Francesco Pierelli

**Affiliations:** 1 Department of Medico-Surgical Sciences and Biotechnologies, Sapienza University of Rome Polo Pontino, Latina, Italy; 2 IRCCS – Fondazione Bietti, Research Unit of Neurophysiology of Vision and Neuro-Ophthalmology, Via Livenza 3, 00198 Rome, Italy; 3 Department of Human Neurosciences, Sapienza University of Rome, Rome, Italy; 4 IRCCS – Neuromed, Pozzilli, IS Italy

**Keywords:** Microstructure, Diffusive metrics, White matter, Chronic pain, Migraine, Descending pain control

## Abstract

**Background:**

We investigated intracerebral fiber bundles using a tract-based spatial statistics (TBSS) analysis of diffusion tensor imaging (DTI) data to verify microstructural integrity in patients with episodic (MO) and chronic migraine (CM).

**Methods:**

We performed DTI in 19 patients with MO within interictal periods, 18 patients with CM without any history of drug abuse, and 18 healthy controls (HCs) using a 3 T magnetic resonance imaging scanner. We calculated diffusion metrics, including fractional anisotropy (FA), axial diffusion (AD), radial diffusion (RD), and mean diffusion (MD).

**Results:**

TBSS revealed no significant differences in the FA, MD, RD, and AD maps between the MO and HC groups. In comparison to the HC group, the CM group exhibited widespread increased RD (bilateral superior [SCR] and posterior corona radiata [PCR], bilateral genu of the corpus callosum [CC], bilateral posterior limb of internal capsule [IC], bilateral superior longitudinal fasciculus [LF]) and MD values (tracts of the right SCR and PCR, right superior LF, and right splenium of the CC). In comparison to the MO group, the CM group showed decreased FA (bilateral SCR and PCR, bilateral body of CC, right superior LF, right forceps minor) and increased MD values (bilateral SCR and right PCR, right body of CC, right superior LF, right splenium of CC, and right posterior limb of IC).

**Conclusion:**

Our results suggest that chronic migraine can be associated with the widespread disruption of normal white matter integrity in the brain.

## Background

Migraine is a chronic disease in nature, but with recurrent acute episodic manifestations. Only a small percentage of people with episodic migraine later develop chronic migraine (CM) [1]. Research has identified an association between episodic and CM and abnormalities in the density and thickness of the gray matter, as well as in functional connectivity at rest or following painful stimulation [2]. Although some studies have found anomalies in the microstructure of the white matter (WM) of migraineurs [3–13], two studies reported there to be no remarkable differences in the integrity of WM microstructure between patients with CM and healthy controls (HCs) [14, 15]. However, patients were scanned during prevention and/or medication overuse. Indeed, we cannot rule out the possibility that, as observed in other brain disorders, some drugs could improve WM fiber bundle connectivity or induce additional damage [16, 17].

Of the various techniques used to analyze magnetic resonance imaging (MRI) data, diffusion tensor imaging (DTI) can be used to effectively examine the microstructural integrity of WM. In this study, we performed tract-based spatial statistics (TBSS) using DTI obtained on a 3 T scanner from patients with episodic and CM who did not have any previous history of medication overuse and were not taking preventive medications; the findings were compared to those obtained from a group of HCs. Furthermore, we extrapolated several diffusivity metrics across all WM from the DTI data, such as fractional anisotropy (FA), mean diffusivity (MD), axial diffusivity (AD), and radial diffusivity (RD), which we further regressed with the patients’ clinical features.

## Methods

### Participants

In accordance with the diagnostic criteria of the International Classification of Headache Disorders (ICHD-III), we prospectively recruited 37 patients with migraine: 19 patients with episodic migraine without aura (MO) and 18 patients with CM. All patients underwent a series of neuroimaging tests. None of the patients enrolled had been prophylactically treated for migraine for at least 3 months, and the patients with CM had no history of excessive use of symptomatic. All patients were tested during the interictal period, which was defined as the period at which it had been at least 3 days since the last attack and 3 days before the subsequent attack for the MO group; only two patients with CM had mild headache at the time of scanning. The exclusion criteria included the presentation of any other neurological disorder, diabetes, high blood pressure, autoimmune or connective diseases, medically-treated major depression, or other types of primary headache. The patient groups were compared to a group of 18 HCs with no personal or family history of migraine and no apparent systemic or neurological disorders. The female participants in this study were tested outside the menstrual cycle. Recording sessions were performed in the afternoon, between 4 and 7 pm. All participants received a complete description of the study and provided written informed consent. The ethical review board of the Faculty of Medicine, University of Rome La Sapienza, Italy, approved the study.

### Image protocols

All participants were scanned using a 3 T Siemens scanner (Verio, Siemens Medical System, Erlangen, Germany) at the “Umberto I” Hospital MR Research Center, Sapienza University, Rome (Italy).

DTI images were obtained with a single-shot echo-planar image sequence with the following parameters: repetition time (TR) = 9300 ms, echo time (TE) = 88 ms, field of view (FOV) = 192 mm × 192 mm, matrix = 96 × 96, 2 mm × 2 mm in-plane resolution, slice thickness = 2 mm, 72 continuous axial slices with no gap, b = 0 s/mm^2^, and b = 1000 s/mm^2^, and 30 diffusion directions were isotropically distributed on a sphere where one direction lacked diffusion weighting.

Structural anatomic scans were performed using T1-weighted sagittal magnetization-prepared rapid gradient echo (MP-RAGE) series (TR = 1900 ms, TE = 2.93 ms, 176 slices, 0.508 × 0.508 × 1 mm^3^ voxels).

We acquired an interleaved double-echo Turbo Spin Echo sequence proton density and T2-weighted images, and their parameters were as follows: TR = 3320 ms, TE = 10/103 ms, FOV = 220 mm × 220 mm, matrix = 384 × 384, slice thickness = 4 mm, gap = 1.2 mm, 50 axial slices.

### DTI image analysis

Before pre-processing, all the DTI image volumes were visually inspected to screen for noisy artifacts due to cardiac pulsatility, signal dropout, and motion artifacts by an expert neuroradiologist (ET). The diffusion images were processed using the Oxford Center for Functional MRI of the Brain’s (FMRIB) Software Library (FSL version 5.0.10, https://fsl.fmrib.ox.ac.uk/fsl). The FSL Diffusion Toolbox was used to correct the eddy currents [18] and motion artifacts [19], while the brain extraction tool was used to create brain masks from the b0 image of each participant [20]. The FSL toolbox DTIFIT fits the pre-processed image based on a diffusion tensor model to yield FA, MD, AD, and RD values.

TBSS was used to conduct the FA voxel-wise statistical analysis according to the following steps [21]: Briefly, each participants’ FA image was registered to a standard space (a 1-mm isotropic FA image [FMRIB58_FA]) with the non-linear registration tool FNIRT (FMRIB’s Nonlinear Registration Tool). A mean FA image was calculated based on all the participants’ images, which were then thinned to obtain the center of major WM tracts common to all subjects with an FA threshold of > 0.2. To avoid misalignment during registration, each participant’s aligned FA map was projected onto the nearest relevant tract center of the mean FA skeleton by searching perpendicular to the local skeleton structure. One skeleton was created using all participants to analyze group differences. We assessed the other three DTI metrics using the same steps used to analyze the FA; the FA, MD, AD, and RD would thus provide more information about the different neural mechanisms of migraine. Based on non-parametric testing (Randomise v2.9), FSL’s permutation was applied to compare the CM, MO, and HC groups.

**Fig. 1:**
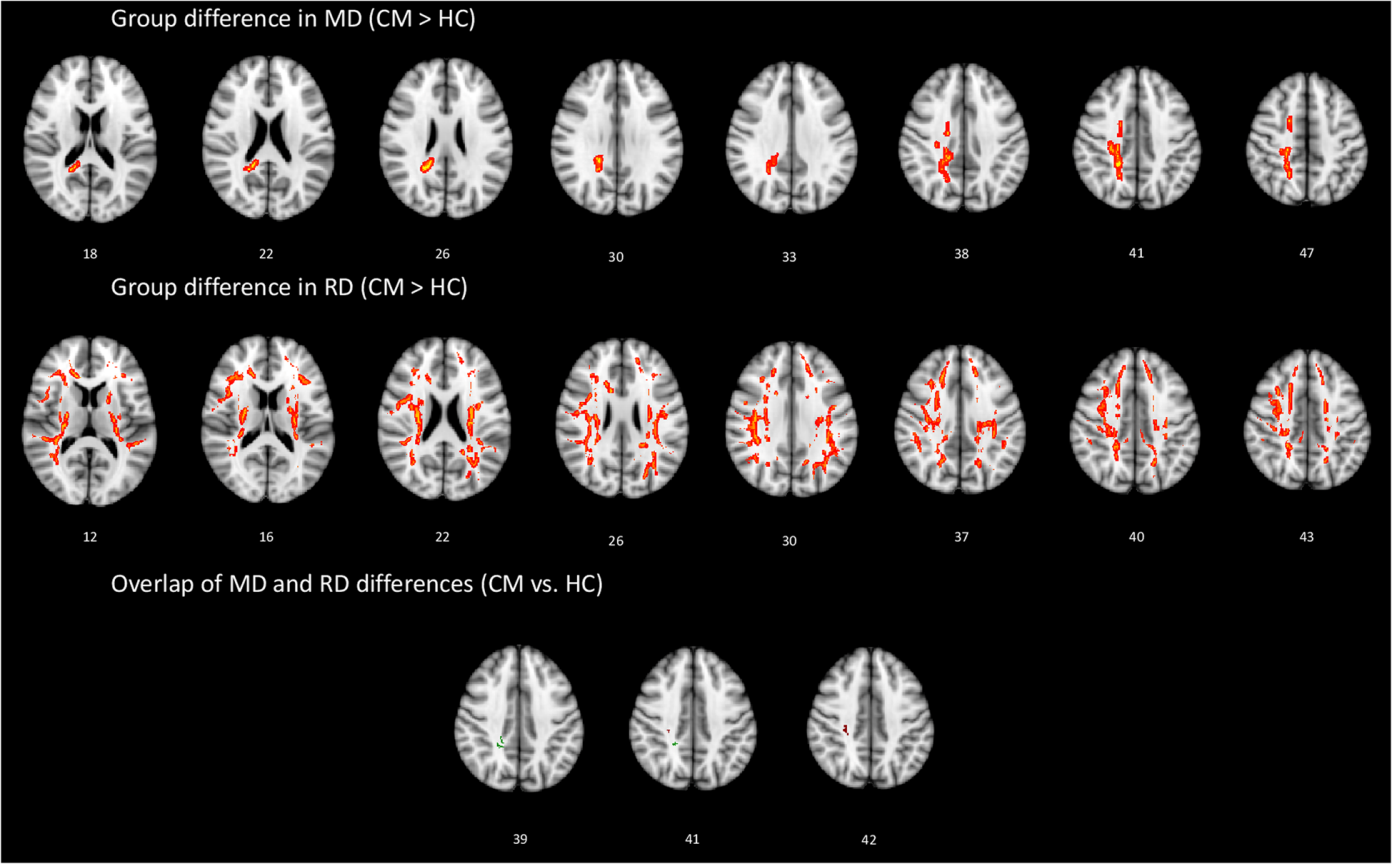
White matter (WM) regions showing increased mean diffusivity (MD) and increased radial diffusivity (RD) in patients with chronic migraine (CM) compared to healthy controls (HC). WM regions showing overlapping MD and RD value differences in the right posterior corona radiata and right superior corona radiata

**Fig. 2:**
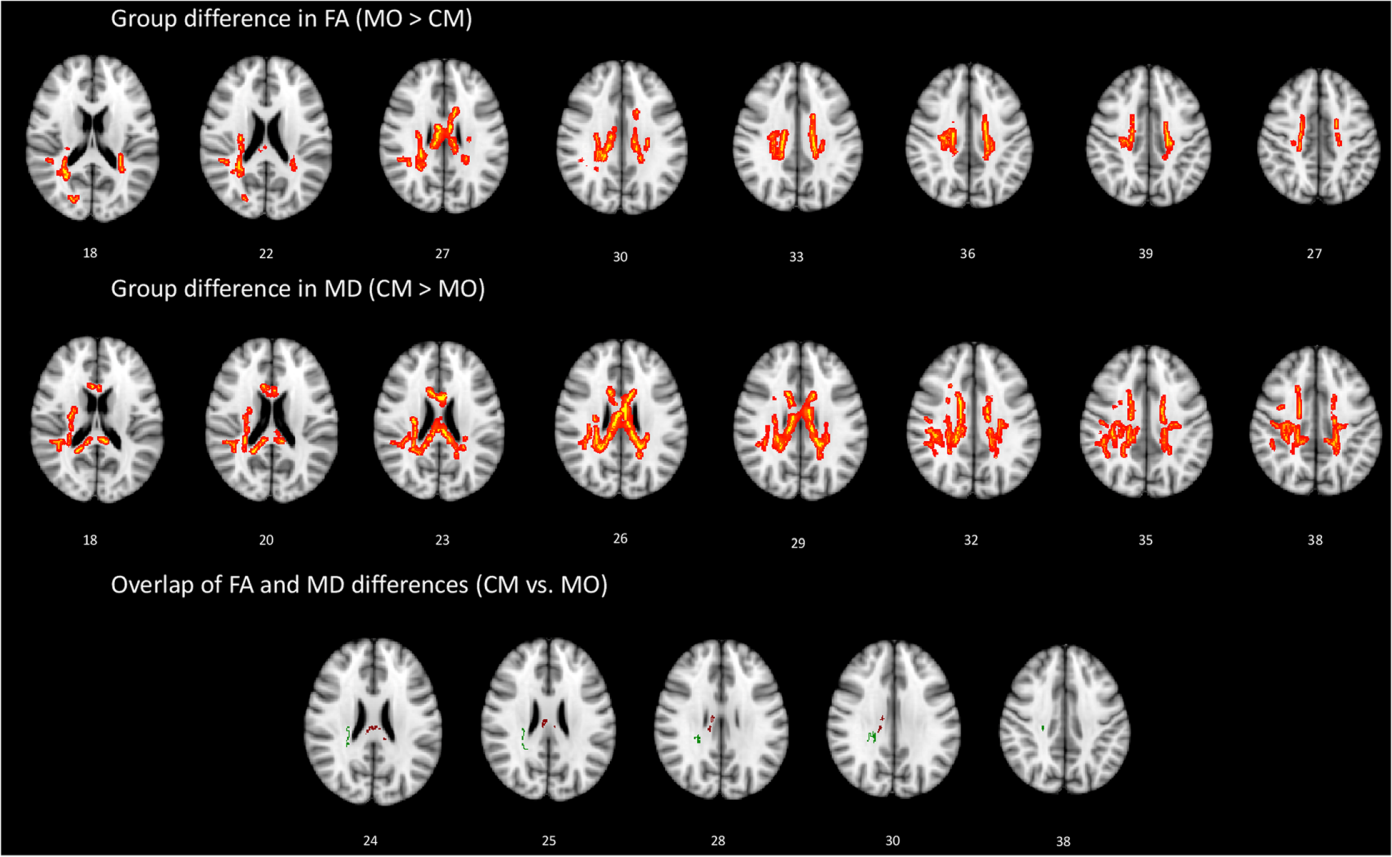
White matter (WM) regions showing decreased fractional anisotropy (FA) and increased mean diffusivity (MD) in patients with chronic migraine (CM) compared to patients with episodic migraine without aura (MO). WM regions showing overlapping FA and MD value differences in the right corpus callosum and right posterior corona radiata

We performed TBSS to conduct six t-contrasts between the three groups using age and sex as covariates. Multiple comparisons were corrected using the threshold-free cluster enhancement method at *p* < 0.05 with a cluster size of > 100 voxels [22]. Contrast maps (Figs. 1 and 2) showed statistically different tracts using the “Tbss_fill” function thresholded at *p* > 0.95. Moreover, the function “cluster” was only used to consider clusters size of > 100 voxels [23]. The template JHU ICBM-DTI-81 WM label atlas was used to identify the anatomical location of the WM tracts that showed significant between-group differences.

Pearson’s correlation analysis was used to evaluate the relationship between the DTI metrics of each CM and MO patients’ tracts with significant between-group differences in the overlapping maps and clinical characteristics (duration of migraine history [years], severity of headache attacks [0–10 Visual Analogue Scale (VAS) score], days from the last migraine attack for MO [n], monthly days with headache and migraine [n], duration of chronic phase [months], and monthly number of acute medication intake [n]). For the correlation analysis, we selected the following regions of interests (ROIs) of MD-RD (for CM vs HC) and of FA-MD (for CM vs MO) overlapping maps, which included WM tracts showing between-group differences in DTI measures: posterior corona radiata R, superior corona radiata R of the CM group for the CM vs. HC comparison, and the superior longitudinal fasciculus R and posterior corona radiata R of the MO and CM groups for the MO vs. CM group comparison (total number of ROIs = 4). A *P*-value of < 0.05/N was chosen to indicate significance (Bonferroni-corrected for multiple comparisons; N corresponds to the number of WM tracts tested) [23].

## Results

All participants completed the study. The two groups of patients and the HC group did not differ in their demographic characteristics, including age (*F* = 1.25, *p* = 0.294) and sex (Chi^2^ = 0.229, *p* = 0.892; Table 1). Structural MRI revealed that none of the study participants had WM lesions. TBSS data of the MO group did not differ from those of the HCs in any of the diffusivity parameters (FA, MD, RD, and AD).

**Table 1:** Clinical and demographic characteristics of healthy controls (HC), migraine without aura patients scanned between attacks (MO) and chronic migraineurs (CM). Data are expressed as the mean ± SD

*Characteristics*	HC (*n* = 18)	MO (*n* = 19)	CM (*n* = 18)
Female (n)	11	13	11
Age (years)	28.4 ± 4.1	31.9 ± 2.1	31.5 ± 10.1
Duration of migraine history (years)		14.2 ± 6.7	14.8 ± 12.8
Attack frequency/month for MO and Headache days/month for CM (n)		3.2 ± 2.1	23.0 ± 7.3
Migraine days/month for CM (n)			14.9 ± 6.3
Days from the last migraine attack for MO (n)			21.0 ± 18.3
Severity of headache attacks (0–10 VAS score)		7.5 ± 0.8	7.4 ± 0.6
Number of acute medication intake/month (n)		2.8 ± 3.1	2.1 ± 0.4

In comparison with the HCs, the CM group exhibited altered diffusivity metrics in many brain regions (Table 1). Specifically, the CM group had increased RD in the WM tracts of the bilateral superior (SCR) and posterior corona radiata (PCR), bilateral genu of the corpus callosum (CC), bilateral posterior limb of the internal capsule (IC), and bilateral superior longitudinal fasciculus (LF), and increased MD values were observed in the tracts of the right SCR and PCR, right superior LF, and right splenium of the CC (Fig. 1). The CM and HC groups featured overlapping WM tracts that indicated increases in MD and RD in the right PCR and right SCR. There were no significant correlations between the areas of overlap or clinical variables.

When compared the MO and CM groups; the CM group exhibited decreased FA values in the tracts of the bilateral SCR and PCR, bilateral body of the CC, right superior LF, and right forceps minor (part of the genu of the CC), and increased MD values in the tracts of the bilateral SCR and right PCR, right body of the CC, right superior LF, right splenium of the CC, and right posterior limb of IC (Fig. 2).

WM tracts that overlapped between the two patient groups that indicated increases in FA and decreases in MD were located in the right CC and right PCR. Specifically, the FA in the right PCR of the MO group was negatively correlated with the mean monthly duration of migraine attacks (*F* = 6.37, *p* = 0.022, *R*^*2*^ = 27.27%, adjusted *R*^*2*^ = 22.99%, regression equation Duration = 206.7–398 FA). In the CM group, there was a tendency towards a negative correlation of the FA in the right PCR with the severity of perceived migraine headache (*F* = 4.35, *p* = 0.05, *R*^*2*^ = 21.36%, adjusted *R*^*2*^ = 16.44%, regression equation VAS = 13.90–13.83 FA). In the MO group, the MD in the right PCR was significantly positively correlated with the severity of perceived migraine headache (*F* = 8.57, *p* = 0.009, *R*^*2*^ = 33.52%, adjusted *R*^*2*^ = 29.61%, regression equation VAS = − 5.64 + 16,921 MD). There were no other significant correlations.

## Discussion

This study found widespread differences in the organization of WM between patients with CM and HCs. In addition, the diffusive metrics of episodic migraineurs, which did not differ significantly from those of HCs, also differed significantly from those of patients with CM.

Among the various techniques used to acquire MRI data, DTI is the most sensitive to microstructural changes by estimating the diffusivity of water molecules along the fiber bundles of WM in the three main orthogonal directions of diffusion. DTI metrics include FA, MD, AD, and RD. FA measures the fraction of the magnitude of the anisotropy; hence, it closely reflects the integrity of the membranes of the WM axons and directionality of the translational movement of the water molecules [24]. MD, which is comprised of radial and axial diffusivity and typically changes in opposition to FA, reflects the overall magnitude of water diffusion by indicating both cellular swelling and cellular density [24]. AD and RD diffusivity are considered to be in vivo surrogate markers of myelin and axonal damage, respectively. These metrics are closely associated with brain microstructure maturation processes as well as with higher levels of cognitive functions [25].

Several studies have shown plastic changes in WM microstructure during the interictal period in patients with migraine. Using a hypothesis-driven approach and a ROI analysis, most of this previous research was performed in patients with episodic forms of migraine; the authors found that – relative to HCs – patients with migraine showed altered DTI metrics in the thalami [10, 13], trigeminal somatosensory pathway [5], genu and the splenium of the CC [11], WM subjacent to area V3A and MT+, left lateral geniculate nucleus [4], and the optic radiations [6]. Whole-brain analyses have revealed contradictory results; indeed, both aberrant diffusive metrics [7, 12, 15, 26, 27] and no alterations [8, 9, 28] have been described in episodic migraineurs without aura when compared to controls. In agreement with the authors who did not detect microstructural abnormalities in patients with MO, we found no differences in the various metrics of diffusivity between patients with MO and HCs. These conflicting findings could be explained by the hypothesis that microstructural anomalies in patients with MO are of an insufficient size to be detected in a stringent statistical comparison with patients with CM, who feature prominent WM abnormalities. As highlighted by a few studies [9, 28], differences in acquisition and analysis parameters could also account for divergent findings.

Previous studies have failed to identify significant differences in DTI metrics between patients with CM and HCs. Part of the controversy can be ascribed to the inclusion of patients receiving preventives and/or overusing medication in a few studies, which could have altered the spontaneous course of the disease and thus biased the results [14, 15]. However, in one study, despite not having identified any significant anomalies in the CM population, the DTI metrics of patients with CM differed significantly from those with MO in several brain regions, including some whose microstructures were found to differ significantly between patients with CM and HCs in the present study [15]. Of such alterations, this study found the most prominent to be in the SCR and PCR, posterior limb of the IC, genu and splenium of the CC, and superior longitudinal fasciculus. These differences were especially evident when RD and MD diffusive metrics were considered. Furthermore, almost the same WM fiber tracts were detected to differ – albeit to a minor extent – between the patients with CM and those with MO, as revealed by a comparison of the corresponding FA and MD values.

The increases in the RD or MD DTI values in the CM group relative to the HC group may indicate axonal abnormalities and decreased cellular density in the WM of patients with CM. On the other hand, the decreased FA and increased MD DTI values of the CM group relative to the MO group might indicate a relatively more severe loss of directional organization and decreased cell density in patients with CM [29]. Moreover, since the swelling of glial cells (especially oligodendrocytes and fibrous astrocytes) could underly the FA changes [30], the morphological changes of glial cells in WM could also account for the altered FA values and, indirectly, MD values of patients with CM. Interestingly, bidirectional aberrant neuro-glial signal transduction has recently been proposed to be a key mechanism underlying chronic pain in general [31], and a genome-wide association study found an association between a gene expressed in glial cells and migraine [32].

Overall, most of the tracts found to exhibit abnormal WM diffusive metrics in the present study form parts of the widespread WM bundle fiber system of the brain that conveys information about somatosensory, cognitive, and/or emotional components of orofacial pain discrimination [33]. Other tracts, like the superior LF, seem to be involved in endogenous pain modulatory mechanisms and multisensory integration [34]. CM is associated with the inability to inhibit cognitive interference [35, 36], low cognitive reserve, and multisensory attention focusing, as well as cognitive deficits in multiple tasks – regardless of the use of medication or the presence of comorbidities – including verbal fluency, spatial dysfunction, and memory retrieval [37]. Thus, patients with CM may be less able to adopt appropriate coping strategies when confronted with daily or almost daily presentation of pain that limits the main activities of daily living. Nonetheless, the abnormalities in the microstructure of WM fiber bundles in patients with CM provide neuroanatomical evidence to support the hypothesis that dysfunctional central pain modulatory circuits contribute to migraine chronification.

Of note, the presently observed neuroradiological findings are similar to those found in relation to other types of cephalic and extra-cephalic chronic pain, such as trigeminal neuralgia [38], fibromyalgia [39], chronic musculoskeletal pain [40], and irritable bowel syndrome [41]. This consistency further supports the hypothesis that WM fiber bundles mediate pain perception and control, and that chronic pain of any type may account for the widespread disruption of WM integrity in the brain.

The WM fiber bundles found to be microstructurally peculiar in the CM group of the present study interconnect numerous and widespread cortical areas, many of those in previous functional resting-state MRI studies in patients with CM, have shown an altered connectivity at rest, including cortical areas belonging to the default, salient, and executive control networks, and their connectivity with the descending pain control system [42–45]. Indeed, in a previous study using the same cohort of patients, we found distinct abnormal connectivity patterns between frontal executive, dorsal attentional, and prefrontal-parietal default networks in patients with CM, as well as a correlation between such structural aberrations and headache severity [46, 47]. The headache severity was also related to the DTI metrics in the present study; the subjective perception of headache intensity was negatively correlated with FA and positively correlated with MD in the overlapping WM tracts of the right PCR in patients with CM and MO, respectively. Since the PCR forms part of the central pain modulatory circuit, and because a similar correlation was previously found in other chronic pain disorders [38, 41], it is possible that an abnormal microstructural integrity of the PCR compromises the perception of pain severity. However, since this was not a longitudinal study, we cannot exclude the possibility that, conversely, the intensity of the pain itself caused the microstructural integrity anomalies. Future studies using the same TBSS method are necessary to follow patients after preventive treatment, to investigate whether abnormal neuroradiological findings can return to normal in CM.

This study was subject to several limitations. First, we collected MRI data in only 30 diffusivity directions. This may have resulted in an underestimation of the degree of WM anomalies in our patients, especially those with MO. Second, the small sample size of the study may have biased our results; however, this limitation was not easy to overcome considering the difficulty of recruiting patients with pure CM and without preventive treatment or a history of drug abuse. Third, due to the lack of longitudinal data, it remains unclear whether the presently observed aberrant WM fiber bundle profiles in patients with CM is the consequence of migraine chronification or an abnormal brain maturation process manifesting early in life – as was shown in previous studies of pediatric migraine patients [48]. Fourth, we did not perform a cognitive assessment of these patients, which would have allowed us to better infer a causal relationship with the microstructural results. Finally, since psychiatric comorbidity and in particular chronic anxiety and depression, even if low-grade and untreated, could affect brain structure, further studies are needed to determine whether this type of comorbidity may have contributed to the microstructural abnormalities detected here.

## Conclusions

We have shown widespread reorganization of WM in patients with CM without medication overuse compared with patients with MO and HCs. In addition, the diffusive metrics of patients also correlated significantly with the subjective perception of headache intensity. These peculiar microstructural pattern of CM patients may indicate axonal abnormalities, decreased cellular density, and/or loss of directional organization in the WM fiber bundles. Further studies are needed to verify whether this reorganization of WM of the CM brain can be normalized by pharmacological and non-pharmacological interventions.

## Data Availability

Not applicable.

## Abbreviations

AD: Axial diffusion
CC: Corpus callosum
CM: Chronic migraine
DTI: Diffusion tensor imaging
FA: Fractional anisotropy
HC: Healthy control
IC: Internal capsule
ICHD: International Classification of Headache Disorders
LF: Longitudinal fasciculus
MD: Mean diffusion
MO: Episodic migraine without aura
PCR: Posterior corona radiata
RD: Radial diffusion
SCR: Superior corona radiata
TBSS: Tract-based spatial statistics
VAS: Visual analogue scale
WM: White matter
